# SEOM-GEIS clinical guideline for gastrointestinal stromal tumors (2022)

**DOI:** 10.1007/s12094-023-03177-7

**Published:** 2023-05-02

**Authors:** César Serrano, Rosa Álvarez, Juan Antonio Carrasco, Gloria Marquina, Jerónimo Martínez-García, Virginia Martínez-Marín, María Ángeles Sala, Ana Sebio, Isabel Sevilla, Javier Martín-Broto

**Affiliations:** 1 Sarcoma Translational Research Group, Vall d’Hebron Institute of Oncology (VHIO), Hospital Universitario Vall d’Hebron, Vall d’Hebron Barcelona Hospital Campus, C/Natzaret, 115-117, 08035 Barcelona, Spain; 2 Hospital General Universitario Gregorio Marañón, Madrid, Spain; 3 Hospital Álvaro Cunqueiro–Complejo Hospitalario Universitario de Vigo, Pontevedra, Spain; 4 Hospital Universitario Clínico San Carlos, Madrid, Spain; 5 Hospital Universitario Virgen de la Arrixaca, Murcia, Spain; 6 Hospital Universitario la Paz, Madrid, Spain; 7 Hospital Universitario Basurto, Bilbao, Spain; 8 Hospital de la Santa Creu I Sant Pau, Barcelona, Spain; 9 Hospitales Universitarios Regional y Virgen de la Victoria, Málaga, Spain; 10 Hospital Universitario Fundación Jiménez Díaz, Madrid, Spain

**Keywords:** GIST, Imatinib, Ripretinib, Avapritinib

## Abstract

Gastrointestinal stromal tumor (GIST) is the most common malignant neoplasm of mesenchymal origin, and a paradigmatic model for a successful rational development of targeted therapies in cancer. The introduction of tyrosine kinase inhibitors with activity against KIT/PDGFRA in both localized and advanced stages has remarkably improved the survival in a disease formerly deemed resistant to all systemic therapies. These guidelines are elaborated by the conjoint effort of the Spanish Society of Medical Oncology (SEOM) and the Spanish Sarcoma Research Group (GEIS) and provide a multidisciplinary and updated consensus for the diagnosis and treatment of GIST patients. We strongly encourage that the managing of these patients should be performed within multidisciplinary teams in reference centers.

## Incidence and epidemiology

Although GISTs are regarded as a rare cancer, they are the most common malignant neoplasm of mesenchymal origin, with an average incidence of 0.4–2 cases per 100,000 inhabitants per year [1].

The stomach is the most frequent location (50–60%), followed by the ileum and jejunum (20–30%), duodenum (3–5%), rectum (2–4.4%), and other locations (< 2%). Cases of extra-gastrointestinal GISTs are exceptional [2].

GIST typically occurs in adults, with a mean age at diagnosis of 60–65 years, and are equally common in male and female patients. Pediatric GISTs are rare, occur at a mean age of diagnosis of 15 years, and have different clinical and molecular features. They are twice as frequent in females than in males, have a multicentric gastric location, and have possible lymph-node metastases. In addition, these patients have a genetic predisposition to the neoplasm frequently related to mutations in the four genes encoding the subunits of the succinate dehydrogenase (SDH) enzyme complex [3].

GISTs are sporadic neoplasms, although infrequently they can appear associated with some inherited conditions, such as Carney–Stratakis syndrome (mutations in SDH subunits), neurofibromatosis type 1, and families with autosomal dominant germline mutations in KIT or PDGFRA [4].

## Methodology

This guideline is based on a systematic review of the most relevant published studies on GIST and is the result of the consensus of ten oncologists with expertise in their management from the GEIS (Spanish Sarcoma Research Group) and the SEOM (Spanish Society of Medical Oncology) and of an external review panel of two experts appointed by the SEOM. The Infectious Diseases Society of America-US Public Health Service Grading System for Ranking Recommendations in Clinical Guidelines has been used to assign levels of evidence (I–V) and grades of recommendation (A–C) [5].

This updated version of the previous SEOM guidelines on GIST describes this tumor's standard diagnostic and therapeutic procedures [4]. A summary of all recommendations is provided in Table 1.

**Table 1:** Therapeutic recommendations and level of evidence

Recommendation	Level of evidence
Diagnostic work-up	
Contrast-enhanced CT-scan is indicated for evaluating tumor extension	III, A
A core needle biopsy is recommended for the initial diagnosis	III, A
It is strongly recommended to perform the mutational analysis in all GIST cases requiring medical treatment	II, A
Performing a biopsy in imatinib-resistant GIST patients with the only objective of the determination of KIT/PDGFRA genotype is not recommended	II, D
There are no validated data supporting the use of circulating tumor DNA for clinical purposes	III, C
Localized disease	
Use of the NIH modified risk criteria to determine the risk of relapse for the indication of adjuvant imatinib	I, A
Imatinib 400 mg daily for a 3-year period is the standard adjuvant treatment in imatinib-sensitive high-risk GIST	I, A
Imatinib 400 mg once daily is acceptable for the adjuvant treatment of *KIT* exon 9-mutant GIST patients	IV, B
It can be considered the use of adjuvant imatinib in intermediate-risk patients with *KIT* exon 11 mutations involving the codons 557 and/or 558	III, B
Adjuvant imatinib is contraindicated in GIST patients with molecular subtypes known to be resistant to imatinib	II, E
Neoadjuvant imatinib can be considered in certain cases with high volume, need of a function-sparing surgery, or risk of bleeding	II, B
Metastatic disease	
Surgery in metastatic disease can be considered on an individual basis within a multidisciplinary tumor board	IV, C
Imatinib 400 mg daily is the standard first-line treatment in metastatic GIST	I, A
Imatinib 800 mg (400 mg/12 h) is preferable in GIST patients with *KIT* exon 9 mutation	II, B
Imatinib treatment should be continued indefinitely until disease progression or drug intolerance	I, A
When disease progresses at the dose of 400 mg/day, an increase to 800 mg/day (400 mg/12 h) is an option	II, B
Continuous dose of sunitinib 37.5 mg once daily can be considered given its better tolerance	III, B
Regorafenib 160 mg daily 3 weeks on, 1 week of is the standard third-line treatment	I, A
Ripretinib 150 mg daily is the standard fourth-line treatment	I, A
Avapritinib 300 mg daily is recommended for the treatment of GIST patients with the PDGFRA D842V, regardless of the line of treatment	III, A

## Diagnosis, pathology, and molecular biology

### Diagnostic evaluation

Gastrointestinal bleeding and abdominal pain are the most frequent symptoms at diagnosis. Bleeding can be chronic (anemia) or acute (hematemesis or melena) requiring an urgent intervention. Additionally, presentation can be as acute abdomen due to tumor rupture or small bowel perforation. In some cases, presentation is asymptomatic [6, 7].

GISTs are generally diagnosed with upper endoscopy (gastric/duodenum locations). Upper endoscopic ultrasound (EUS) is useful for the detection of small intramural lesions. Abdomen–pelvis contrast-enhanced computerized tomography (CT) scan with image acquisitions of the arterial and portal phases is indicated for evaluating tumor extension (III, A). GIST rarely metastasizes to thorax, so thorax CT-scan is not routinely recommended. MRI is the best imaging technique for rectum GIST or for characterizing uncertain liver lesions [I, A]. Positron Emission Tomography (PET)-CT is not routinely recommended, unless early prediction of response to first-line imatinib is anticipated during the initial work-up [8]. Serum tumoral markers are not required for GIST diagnosis [9]. A core- (preferable) or a fine-needle biopsy can be performed via EUS, and it is enough for the GIST diagnosis prior surgery. However, if radiological images are conclusive or highly suggestive of GIST, surgery can be performed without a prior histological assessment. This is the case of abdominal nodules or masses not amenable of endoscopic assessment and in which an external biopsy can lead to intraperitoneal tumor spillage or rupture [10]. Noteworthy, if neoadjuvant or first-line treatment is primarily indicated, a core needle biopsy is mandatory for diagnosis (III, A) [9]. In some cases, GISTs are smaller than 2 cm and surgical excision could be the only approach for histological diagnosis.

### Pathology

A core needle biopsy is the standard approach for histological and molecular assessment. The pathology evaluation should include the description of morphological features, mitotic count expressed in number of mitoses per 5 mm^2^, and immunohistochemistry (IHQ) determinations. A recommended IHQ panel includes the following antibodies and rates of positivity: CD117 (95%), DOG1 (98%), CD34 (70–90%), actin (20–30%), S100 (8–10%), and desmin (2–4%) [11]. In rare cases with suspicion of GIST diagnosis but negative for CD117 and DOG1, molecular determination of KIT/PDGFRA mutations can be of aid [12].

In surgically resected specimens, the following features should be reported: site and size of the primary tumor, mitosis per 5 mm^2^ in the most proliferative areas, margins, histologic sub-type (spindle cell, 77%; epithelioid, 8%; or mixed, 15%), presence of necrosis, and tumor rupture or perforation. In rare cases of resected lymph nodes under the surgical suspicion of tumoral involvement, their pathological description should be added. The same IHQ panel can be used here. Importantly, Ki-67 immunostain must not replace the mitotic count [11, 12].

### Molecular biology

Between 85 and 90% of GISTs are caused by activating mutations in the KIT or PDGFRA genes [12]. It is strongly recommended to perform the mutational analysis, in either localized or metastatic lesions, when medical treatment is indicated, as these analyses provide prognostic and predictive information for response to approved tyrosine kinase inhibitors (TKIs) (II, A). KIT/PDGFRA molecular studies are commonly performed in paraffine-embedded formalin-fixed tumors through Sanger or Next-Generation Sequencing (NGS). *KIT* primary mutations (75%) often emerge in exons 11 and 9, and less frequently in exons 13 and 17. Although all of them are sensitive to first-line imatinib, *KIT* exon 11-mutants are more sensitive than exon 9, and the latter commonly requires double dose of imatinib for metastatic disease [13]. If *KIT* exon 11 codons 557 and/or 558 are affected, there is a higher risk of relapse in intermediate-risk, surgically resected GISTs [14]. *PDGFRA* mutations are commonly found in exon 18 (5% of all GIST), being less frequent in exons 12 or 14. The *PDGFRA* exon 18 D842V substitution was formerly deemed resistant to all available TKIs until the recent activity shown by avapritinib [15].

Resistance to TKIs in GIST is commonly (> 90%) due to the polyclonal emergence of secondary mutations in KIT or PDGFRA [16]. Thus, routine determination of KIT/PDGFRA genotype is not recommended in imatinib-resistant GIST patients given the heterogeneity of resistance mutations (II, D). Likewise, there are no current validated data supporting the use of circulating tumor DNA to take clinical decisions, although it can be assessed with investigational purposes (III, C) [17].

Between 10 and 15% of all GISTs are wild type (WT) for KIT and PDGFRA mutations. In these cases, IHQ for SDHB can identify SDH-deficient GISTs, which represent the overwhelming majority of KIT/PDGFRA WT GIST. The hereditary implications of SDH-deficient GISTs have been discussed above. Of note, the Carney triad syndrome is a not heritable condition consisting of gastric GISTs, pulmonary chondromas, and paragangliomas. Driver alterations in KIT/PDGFRA WT GIST positive for SDHB are diverse and may affect RAS, BRAF, NF1, and NTRK [12].

### Recommendations for the diagnostic work-up

1. Abdomen–pelvis contrast-enhanced CT-scan with image acquisitions of the arterial and portal phases is indicated for evaluating tumor extension (III, A).
2. A core needle biopsy is recommended for the initial diagnosis (III, A).
3. It is strongly recommended to perform the mutational analysis in all GIST cases requiring medical treatment (II, A).
4. Performing a biopsy in imatinib-resistant GIST patients with the only objective of the determination of KIT/PDGFRA genotype is not recommended (II, D).
5. There are no validated data supporting the use of circulating tumor DNA for clinical purposes (III, C).

## Staging and risk assessment in localized GIST

Relapse-risk assessment for surgically resected primary GIST is critical not only to provide prognostic information, but also to estimate the potential benefit of adjuvant imatinib. Prognostic factors in GIST include mitotic count (expressed as the number of mitoses on a total area of 5 mm^2^), tumor size, and tumor site (extra-gastric location entails worse outcome). Spontaneous or intraoperative capsule rupture should also be recorded and considered as a very poor prognostic factor.

Several risk-stratification systems have been proposed and include some or all the aforementioned prognostic factors. The most validated risk criteria are the Armed Forces Institute of Pathology (AFIP) criteria and the National Institute of Health (NIH) modified risk criteria [2, 18]. We recommend using the latter as it has been the basis for the indication of adjuvant imatinib in all contemporary clinical trials (Table 2) (I, A). More recently, a novel risk classification based on heatmaps is being increasingly used [19]. This system considers the same prognostic factors but as continuous variables. Adjuvant imatinib is usually recommended for those cases with a probability of recurrence higher than 40% using this heatmap model.

**Table 2:** Guidelines for risk assessment of primary GIST: modified NIH consensus criteria

Tumor size (cm)	Mitotic count (/5 mm^2^)	Tumor location
Very low risk		
< 2	≤ 5	Any site
Low risk		
2.1–5.0	≤ 5	Any site
Intermediate risk		
≤ 5.0	6–10	Gastric
5.1–10.0	≤ 5	Gastric
High risk		
> 10.0	Any count	Any site
Any size	> 10	Any site
> 5.0	> 5	Any site
≤ 5.0	> 5	Nongastric
5.1–10.0	≤ 5	Nongastric

## Management of local and locoregional disease

**Fig. 1:**
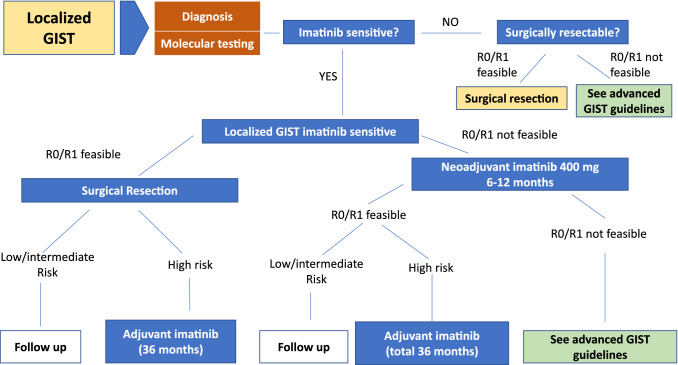
Algorithm for the management of localized disease

The algorithm for the management of localized disease is depicted in Fig. 1.

### Surgery

Approximately 70% of patients with GIST will be cured by surgery alone, and therefore, it constitutes the standard treatment for localized GIST ≥ 2 cm or GISTs with suspicious EUS features [12]. R0 resection (negative margins) with a 1-cm margin is the aim of surgery, although seemingly an R1 surgery (microscopic positive margins) does not involve higher risk of recurrence nor worse survival [20]. Peritoneal or liver spread is exceedingly more common than local recurrence. With the exception of SDH-deficient GISTs, routine local lymphadenectomy is not indicated, although nearby enlarged lymph nodes should be removed [21]. GISTs are prone to bleed, friable, and easy to rupture masses, which is an independent poor prognostic factor [19]. The most common location of GISTs is the stomach and a formal anatomic gastrectomy is rarely required. Conversely, segmental resection with a 1-cm radial margin is the most common approach [22]. Segmental resection is also the best choice in GIST from the small bowel. However, pancreaticoduodenectomy or Whipple resection can be required in duodenal periampullary tumors. Surgery for rectal GISTs with anastomosis to the remaining rectum can be done but in GIST close to the anal sphincter abdominoperineal resection with permanent colostomy is required. Novel transanal techniques to remove rectal GISTs have been described although mostly in case reports.

Laparoscopic surgery is indicated if the GIST is less than 5 cm and up to 8 cm in size can be removed laparoscopically with retrospective data favoring this approach regarding less hospital staying and wound complications [23]. Data from endoscopic resection for small GISTs (most of them < 2 cm) have mixed results, with potential for perforation. Enucleation techniques attempt to preserve organ’s function and can be suitable for polyp-like GISTs with a narrow connection to muscularis propria [12].

In SDH-deficient and NF1-associated GISTs presenting as multifocal disease, surgery is often indicated when one or a few tumors are growing faster than the others or have become symptomatic.

### Adjuvant treatment with imatinib

Three randomized phase III trials established the indication and duration of adjuvant treatment in GIST. The ACOSOG Z9001 trial showed superiority in terms of recurrence-free survival (RFS) for imatinib against placebo in resected GIST tumors larger than 3 cm [24]. Later on, the EORTC 62,024/GEIS-10 evaluated 2 vs 0 years in intermediate- and high-risk GIST patients showing a benefit in imatinib failure-free for treated high-risk [25]. This benefit was not statistically significant, but the observed trend supports the results of the Scandinavian/German trial SSGX-VIII/AIO in which 3 years of adjuvant imatinib proved superior regarding overall survival and RFS in high-risk (following NIH modifications) GIST patients [26]. After 10-year follow-up, overall survival at 10 years was 79% in the 3-year arm compared to 65.3% in the 1-year arm [27].

The optimal duration of the adjuvant treatment is still under evaluation. In the single-arm phase II trial PERSIST, no patient with an imatinib-sensitive GIST recurred during the 5-year treatment period [28]. Currently, a phase III randomized trial is evaluating 3- vs 5-year imatinib in high-risk GIST (NCT02413736).

Based on the previous studies, to date, standard adjuvant treatment for surgically resected, imatinib-sensitive high-risk GIST consists on imatinib 400 mg daily for a 3-year period (I,A).

Molecular testing for KIT and PDGFRA is mandatory if adjuvant treatment is indicated (II, A). Data from the metastatic setting, the MetaGIST metaanalysis, showed that *KIT* exon 9-mutant GISTs are more sensitive to the 800 mg dose [29]. However, no data support this practice in the adjuvant setting and the only available retrospective series suggests that the use of 400 mg daily already provides benefit in this molecular subset (IV, B) [30]. For intermediate-risk patients, molecular testing might help to tip the balance toward adjuvant treatment if KIT exon 11 557–558 codons are affected (III, B) [14]. Patients with imatinib-resistant mutations, such as PDGFRA D842V, should not receive adjuvant treatment (II, E) [15]. The same principle applies for all WT GIST (IV, D) (II, E) [12].

### Neoadjuvant treatment with imatinib

Neoadjuvant treatment can be considered in imatinib-sensitive GIST patients (II, B). Patients benefiting from preoperative treatment include those for which an R0 resection cannot be initially obtained, patients in need of a function-sparing surgery (i.e., rectal GIST), or those considered at risk of bleeding or tumor rupture in which prior imatinib will reduce these risks [31]. Molecular testing is mandatory before its initiation. Patients should be followed up closely at the beginning to confirm the benefit of imatinib. Surgery is commonly undertaken between 6 and 9 months after treatment initiation. The completion of 3 years of adjuvant imatinib after the surgical procedure is based on the risk criteria assessed by the CT-scan (size, location) and the tumor biopsy (mitotic count) prior imatinib initiation.

### Recommendations for the management of localized disease

1. Use of the NIH modified risk criteria to determine the risk of relapse for the indication of adjuvant imatinib (I, A).
2. Standard adjuvant treatment for surgically resected, imatinib-sensitive high-risk GIST consists of imatinib 400 mg daily for a 3-year period (I, A).
3. Imatinib 400 mg once daily is acceptable for the adjuvant treatment of *KIT* exon 9-mutant GIST patients (IV, B).
4. Clinicians might consider the use of adjuvant imatinib in intermediate-risk patients with *KIT* exon 11 mutations involving the codons 557 and/or 558 (III, B).
5. Adjuvant imatinib is contraindicated in GIST patients with molecular subtypes known to be resistant to imatinib (II, E).
6. Neoadjuvant imatinib can be considered in certain cases with high volume, need of a function-sparing surgery, or risk of bleeding (II, B).

## Management of advanced and metastatic disease

**Fig. 2:**
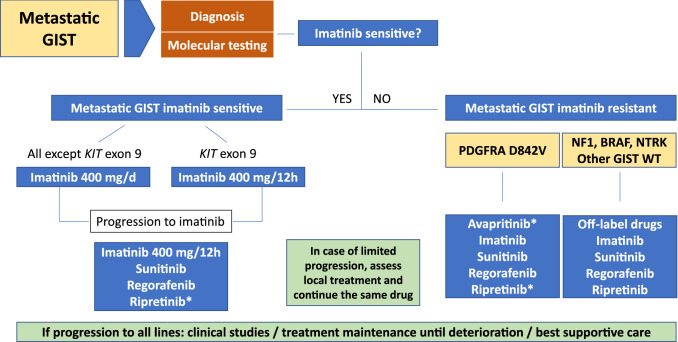
Algorithm for the management of metastatic disease

The algorithm for the management of metastatic disease is depicted in Fig. 2.

### Surgery and other local treatments for metastatic disease

Surgery for metastatic GIST can be discussed by an expert team within a multidisciplinary tumor board. On the one hand, it can be indicated as a debulking procedure during imatinib response to reduce the number of resistant clones and delay tumor progression (IV, C). Several series have suggested its positive impact in overall survival, although all these studies were retrospective and thus likely biased [32]. On the other hand, surgery can be also considered in the case of unifocal progression (IV, C). This approach can benefit mainly patients on imatinib [33]. However, it can be considered in later lines depending on the time on drug. As all these patients have uncurable metastatic disease, the same TKI used before the surgical procedure must be continued after the resection, regardless of the type of procedure—cytoreductive surgery or resection of resistance unifocal progression.

Finally, the evidence for other approaches, such as embolization or radiofrequency, is lacking, and therefore, surgery should be prioritized. Nonetheless, these patients are metastatic, and therefore, it is important to avoid mutilating procedures, which in turn may leave some room for these alternative procedures discussed on an individual basis by multidisciplinary teams in sarcoma-expert centers (IV, D).

### KIT- and PDGFRA-mutant GIST

#### First-line treatment with imatinib

Imatinib, at 400 mg daily, is the standard treatment in locally advanced unresectable, and in metastatic GIST patients. This evidence is based on the results from two randomized phase III trials (I, A) [34, 35]. The median PFS for patients treated with imatinib is 20–24 months, with a clinical benefit rate of 88%. Imatinib is also the standard treatment for those patients with completely resected metastatic disease, and in GIST patients that develop metastatic disease after the finalization of adjuvant imatinib. Tumor genotyping before starting imatinib therapy is highly recommended, as it predicts outcomes and guides treatment decisions (II, A) [36]. Hence, the first-line treatment for patients with *KIT* exon 9 mutation is 400 mg twice daily of imatinib (II, B), obtaining a significantly higher response rate and improved mPFS [29]. Likewise, imatinib seems ineffective in certain genotypes, such as the *PDGFRA* exon 18 D842V mutations and in KIT/PDGFRA WT GIST [12, 15]. The activity of imatinib of imatinib in infrequent genotypes (i.e., *KIT* primary exons 13 or 17 mutations and other PDGFRA mutations) is poorly understood, but preclinical studies deem these alterations as imatinib-sensitive [36, 37].

Imatinib is often well tolerable at the 400 mg daily dose. The most common adverse events are edema (70%), mostly periorbital, nausea (50%), diarrhea (45%), myalgia (40%), fatigue (35%), dermatitis or erythema (30%), headache (25%), and abdominal pain (25%).

Imatinib treatment should be continued indefinitely until disease progression or drug intolerance (I, A). Outside of these two assumptions, the treatment must not be suspended as virtually all patients relapse [38]. However, between 5 and 10% of all metastatic GISTs achieve durable responses with imatinib (i.e., > 10 years) [39]. As there is no demonstration that these patients are cured, imatinib should not be discontinued regardless of the treatment duration. Special caution should be given to kidney function, as long-term imatinib treatment can be associated with drug-induced kidney failure [40].

#### Systemic treatment following imatinib failure

Imatinib achieves clinical benefit, to a greater or lesser extent, in all cases with imatinib-sensitive mutations. However, the great majority of GIST patients will develop secondary resistance with a median time to progression of about 24 months. All clinical data, including lesion density on CT-scan, potential drug interactions, and treatment compliance, should be assessed prior to dose escalation of imatinib or switching to sunitinib, the two alternatives after failure to first-line imatinib 400 mg daily.

When disease progresses at the dose of 400 mg/day, an increase to 800 mg/day (400 mg/12 h) is an option (II, B). The conjoined analysis of two phase III trials showed maintained partial responses or stable disease for 81 days in 30% of the patients [29]. However, the benefit in molecular subgroups other than *KIT* exon 9-mutant seems marginal.

Sunitinib is an oral multikinase inhibitor with activity against KIT and PDGFRA, among several other kinases. A pivotal phase III study reported a response rate in imatinib-resistant GIST of nearly 10%, with a clinical benefit rate of approximately 65% [41]. The median PFS of 6 months was more than four times longer than that of the placebo arm. Based on these results, sunitinib 50 mg/day on an intermittent dosing schedule of 4 weeks on treatment followed by 2 weeks off received the regulatory approval as the second-line treatment in advanced, imatinib- or imatinib-intolerant GIST (I, A). Asthenia, skin toxicity, diarrhea, hypertension, and hypothyroidism are the most frequent adverse events with sunitinib. Close monitoring of hypertension, cardiac function, and thyroid hormones is indicated during sunitinib therapy. A later single-arm phase II trial with continuous daily dose of 37.5 mg showed comparable activity and better tolerability, thus constituting a valid alternative (III, B) [42].

Regorafenib, is the standard third line approved for the treatment of unresectable and/or metastatic GIST patients after failure of imatinib and sunitinib (I, A). A phase III randomized trial evaluated 28-day cycles of regorafenib 160 mg daily, 3 weeks on, 1 week off, using placebo as the comparator arm. Regorafenib treatment achieved an mPFS of 4.8 months, a clinical benefit rate at 12 weeks of 52.6%, and an overall response rate of 4.5%. The toxicity profile of regorafenib was consistent with that of other kinase inhibitors with similar target spectrum, and the most common adverse events were hypertension, hand–foot skin reaction, and diarrhea [43].

More recently, in November 2021, the European Medicines Agency (EMA) approved the TKI ripretinib as the new fourth-line standard-of-care for the treatment of advanced or metastatic GIST (I, A). This approval is based on the results of the phase III, placebo-controlled, INVICTUS trial [44]. Ripretinib 150 mg once daily showed an mPFS of 6.3 months and an overall response rate of 9.4%. Side effects are overall manageable and consistent with KIT and PDGFRA inhibition, as imatinib. Additionally, alopecia and low-grade hand–foot skin reaction are also frequent. Despite this evidence, ripretinib is still awaiting financial approval from the health authorities in Spain.

Participation in clinical trials should be always considered in GIST, and especially after ripretinib failure, since no standard treatment options are approved at this stage. Other therapeutic options may include cabozantinib, pazopanib, and rechallenge of prior drugs [12].

### Treatment of other GIST molecular subtypes

#### GISTs harboring the *PDGFRA* exon 18 D842V mutation

Approximately 5% of all GIST have the *PDGFRA* D842V missense mutation as the primary driver [15]. Metastatic GIST patients with this mutation are treated similarly to other GISTs despite all TKIs approved have little-to-no activity against this mutation. The phase I NAVIGATOR trial studied the activity of the type I TKI avapritinib in 56 D842V-mutant GISTs, including 11 TKI-naïve. The overall response rate was 91%, the clinical benefit rate of 98%, and mPFS of 34 months, which constitute an unprecedented activity in this molecular subset of GIST [45]. Based on this data, the EMA approved in September 2020 avapritinib for the treatment of metastatic GIST patients with this specific molecular alteration (III, A). Despite this evidence, avapritinib is still awaiting financial approval from the health authorities in Spain. Side effects are manageable and consistent with strong inhibition of KIT and PDGFRA. Most common toxicities are nausea, fatigue, anemia, diarrhea, and edema, and also a characteristic increase in cognitive effects in 37% of the patients that require strict monitoring [46].

#### SDH-deficient GIST

The activity of first-line imatinib in this subset of patients is unknown. However, scattered data suggest that multikinase inhibitors with anti-VEGFR function, such as sunitinib and regorafenib, are effective in these patients (III, B) [47, 48]. However, no therapies are specifically approved for this sub-type. Preliminary data suggest that temozolomide can be a potential option [49].

#### Other molecular drivers in KIT/PDGFRA WT GIST

Few, if any, case reports justify potential treatment alternative for NF1 or BRAF-mutant GIST, such as MEK and/or BRAF inhibitors (IV, B). Some KIT/PDGFRA WT GIST appear to have rearrangements involving NTRK. Two clinical trials showed significant activity in NTRK-fused cancer, including GIST [50]. However, despite the EMA approved these two therapies for the treatment of these types of cancer (III, A), the health authorities in Spain did not grant the approval for these treatments.

### Recommendations for metastatic disease

1. Surgery in metastatic disease can be considered on an individual basis within a multidisciplinary tumor board (IV, C).
2. Imatinib 400 mg daily is the standard first-line treatment in metastatic GIST (I, A).
3. Imatinib 800 mg (400 mg/12 h) is preferable in GIST patients with *KIT* exon 9 mutation (I, B).
4. Imatinib treatment should be continued indefinitely until disease progression or drug intolerance (I, A).
5. When disease progresses at the dose of 400 mg/day, an increase to 800 mg/day (400 mg / 12 h) is an option (II, B).
6. Sunitinib 50 mg daily 4 weeks on, 2 weeks off is the standard second-line treatment (I, A).
7. Continuous dose of sunitinib 37.5 mg once daily can be considered given its better tolerance (III, B).
8. Regorafenib 160 mg daily 3 weeks on, 1 week of is the standard third-line treatment (I, A).
9. Ripretinib 150 mg daily is the standard fourth-line treatment (I, A).
10. Avapritinib 300 mg daily is recommended for the treatment of GIST patients with the PDGFRA D842V, regardless the line of treatment (III, A).

## Follow-up, long-term implications, and survivorship

There are no clinical trials assessing follow-up of patients with GIST. Follow-up recommendations are based on expert opinions and are tailored to the risk of relapse, which depend on tumor localization, size, mitosis, and tumor rupture for localized and resected GIST (IV, C). The aim of follow-up in GIST is the potential for early detection of recurrence, when the bulk is still small [51]. Abdominopelvic CT or MRI should be used as relapse usually occurs in peritoneum or liver. Endoscopy is only indicated in familial GISTs and in some cases of R1 resection in gastric, esophageal, or rectal tumors. The recommendation for intermediate–high-risk localized resected patients is to perform a CT-scan every 3–6 months in the first 3 years, then every 6 months up to 5 years, and then annually.

After stopping adjuvant imatinib, a closest follow-up is necessary for the following 2 years, when the risk of recurrence is the greatest, with CT-scan at 3-month intervals [52]. PET/CT may be considered to clarify ambiguous CT results.
